# Names and Things: The Universal Craving for Euphemisms

**Published:** 1916-10-28

**Authors:** 


### NAMES AND THINGS.

### The Universal Craving for Euphemisms.

It is pathetic to witness the efforts of those who practise in mental diseases, and those who have mad friends, to diminish the terrors of madness by giving to it another name. Madness is so objectionable, so terrifying, so repugnant, so degrading, that we admit its existence with reluctance, and when it can no longer be denied or concealed we try to hide it by the childish expedient of calling it something else. It is a Sisyphean labour.

With many a weary step, and many a groan,
Up the high hill he heaves a huge round stone;
The huge round stone, resulting with a bound,
Thunders impetuous down, and smokes along the ground.

No sooner has madness been decently and comfortably hidden away and concealed behind the mask of lunacy, which has the harmless meaning of being governed by the moon, than the antic begins to grin and mowe through the mask, which becomes more and more transparent until before long it ceases to be any concealment, and the horror appears as plain as ever. Lunacy, which at first decently concealed the features of madness, soon itself becomes as terrifying, and a new mask must be found to hide its hideous features. Such a mask is found in insanity, which means merely out of health, and now for a short time the death's head is hidden again; but again the mask soon becomes thin, again the horrid features grin through it, again the terror appears in all its hideous intensity, and a new mask must be found, the stone must again be heaved up the hill, and under the name of unsoundness of mind again it comes tumbling down and smokes along the ground. Unsoundness of mind soon becomes as transparent and flimsy a disguise as insanity and lunacy, and another mask must be clapped over the ugly features, so we now exchange unsoundness of mind for nervous breakdown. This will serve its purpose for a few years, and then the weary task must be undertaken again, once more the stone must be heaved up the hill, once more it will come tumbling down, and a new expedient must be devised, a new name found, a new concealment attempted, only to prove as futile as every previous effort.

We see in other instances the same futile effort to cover up with a verbal cloak what is objectionable, and we see in these instances also how soon the verbal cloak wears thin, becomes transparent, and ceases to have any power of concealment. The privy place or privy of our forefathers superseded a coarser name, and for a short time was a decent mode of referring to what was soon called the necessary house, because the word privy had taken on all the objectionable features of its predecessor. With the introduction of a new method of scavenging, the necessary house, now become as indecent as the privy, became the water-closet, a name perfectly decent at first, but soon to be tabooed as completely as its predecessors, even after it had been abbreviated to its initials, which was the next futile attempt at concealment. These titles had at least the merit that they were descriptive, and truly described the place they referred to, but the next effort produced a false and misleading title. The place is now called a lavatory, which it certainly is not, and when we wish to refer to a lavatory properly so called we are confronted with the certainty that we shall be misunderstood. It would have been much better, if we must have a new name for it, to call it the drawing-room, or withdrawing-room, which is at least truly descriptive, though it shares with lavatory the disadvantage that it has already been given to another kind of apartment.

The same desire to get rid of the objectionable features of a thing by hiding it under a new name led our forefathers to supersede the Devil by the Evil One, who seemed less formidable under his new title. Subsequently he was alluded to as Old Nick, and as the Old Gentleman, and no doubt other appellations would have been invented to conceal his terrors were it not that the terrors themselves have vanished.

Lately we have had proposals to drop the title lunatic asylum, and substitute for it the title mental hospital. It is another Sisyphean attempt to heave the stone to the top of the hill. The wiseacres who make the proposal forget, or more probably are ignorant, that the title lunatic asylum was substituted for madhouse in order to cloak the unpleasantness of this term. An asylum is a refuge, a place to which people fly for security and peace, and no more alluring substitute for madhouse can be devised. The Quakers use retreat for the same purpose, but the inevitable has happened, and asylum and retreat have gathered to themselves all the terrifying meaning of the old madhouse. Mental hospitals will share the same fate. Again the stone so laboriously heaved up the hill will come rolling and slipping down, and our successors will have the same thankless task before them.

Shall we never learn that we cannot alter the character of a thing by altering its name? Savages think they can. They believe that knowledge of the name of a thing gives them power over that thing, and that when they alter its name they alter the thing. The ingenious persons who devise new names for madness and madhouses are in this respect no wiser than savages; nay, they are less wise, for they have behind them a long and varied experience proving the futility and absurdity of their endeavours, and one of these experiences shows them clearly enough, if they had but eyes to see it, the true purpose to which their efforts should be directed. There was a time when people were as chary of mentioning the devil by his proper title as they now are of mentioning madness by its proper title. The name of the devil was taboo, and if it was necessary to refer to him, the reference must be made indirectly, in a roundabout way, by a pseudonym. But no one now thinks it necessary so to refer to him. If we want to speak of the devil, as indeed we seldom do, we have no hesitation in giving him his proper title. Why is this? It is because he is shorn of his terrors. No one but a few old women now believes in the existence of a personal devil, or if they do, they regard him not as a powerful monster going about as a roaring lion seeking whom he may devour, but as a mischievous imp, much as our forefathers regarded Robin Goodfellow. Since he no longer terrorises we may speak of him as we please. The moral is obvious. If we want to find for madness and madhouses an appellation that shall not be frightful, it is no use to alter the appellation from time to time, for the new appellation will soon take on all the terrors of the old. The only way is to divest the thing named of its terrors. As long as madness is regarded with superstitious horror as a visitation of God or as possession by the devil, so long people will shrink from referring to it, whatever name they give to it. As soon as they can be educated into regarding it as a disease of natural origin, no more mysterious or supernatural than tuberculosis or small-pox, the horror will fall from it, and it will be referred to by its right name, without tremor and without shrinking. The silly people who call madness nervous breakdown or neurasthenia, and madhouses mental hospitals, are perpetuating the barbarous view of insanity as demoniacal possession, and are proving their imperfect emergence from savagery by adopting the expedient of the savage. They would be better employed, and would show that they had climbed higher towards civilisation, if they would direct their efforts to teaching that madness is a disease like other diseases, a disease from which no one is immune, a disease that is no more disgraceful than measles, and not nearly as disgraceful as the superstition that a thing can be altered by altering its name, or that a terrible thing ceases to be terrible when it is called by a soothing appellation.

